# Genomic and Immunologic Correlates of Indoleamine 2,3-Dioxygenase Pathway Expression in Cancer

**DOI:** 10.3389/fgene.2021.706435

**Published:** 2021-07-22

**Authors:** Anshuman Panda, Shridar Ganesan

**Affiliations:** ^1^Department of Medical Oncology, Rutgers Cancer Institute of New Jersey, New Brunswick, NJ, United States; ^2^Department of Medicine, Rutgers Robert Wood Johnson Medical School, New Brunswick, NJ, United States

**Keywords:** IDO1 inhibitor, IDO2, TDO2, immune checkpoint inhibitors, CD8^+^ T-cell, tumor mutation burden, virally induced cancer, endogenous retrovirus

## Abstract

Immune checkpoint blockade leads to unprecedented responses in many cancer types. An alternative method of unleashing anti-tumor immune response is to target immunosuppressive metabolic pathways like the indoleamine 2,3-dioxygenase (IDO) pathway. Despite promising results in Phase I/II clinical trials, an IDO-1 inhibitor did not show clinical benefit in a Phase III clinical trial. Since, a treatment can be quite effective in a specific subset without being effective in the whole cancer type, it is important to identify the subsets of cancers that may benefit from IDO-1 inhibitors. In this study, we looked for the genomic and immunologic correlates of IDO pathway expression in cancer using the Cancer Genome Atlas (TCGA) dataset. Strong CD8^+^ T-cell infiltration, high mutation burden, and expression of exogenous viruses [Epstein-Barr virus (EBV), Human papilloma virus (HPV), and Hepatitis C virus (HCV)] or endogenous retrovirus (*ERV3-2*) were associated with over-expression of *IDO-1* in most cancer types, *IDO-2* in many cancer types, and *TDO-2* in a few cancer types. High mutation burden in ER+ HER2− breast cancer, and *ERV3-2* expression in ER− HER2− and HER2+ breast, colon, and endometrial cancers were associated with over-expression of all three genes. These results may have important implications for guiding development clinical trials of IDO-1 inhibitors.

## Introduction

Unleashing anti-tumor immune response by targeting the immune checkpoint pathways (Pardoll, 2012; e.g., PD-1 blockade) leads to remarkable and durable responses in many cancer types (Kyi and Postow, 2016; Hargadon et al., 2018; Arora et al., 2019). An alternative method of unleashing anti-tumor immune response is to target the indoleamine 2,3-dioxygenase (IDO) pathway (Liu et al., 2010; Munn, 2012), a metabolic pathway that suppresses immune response (Frumento et al., 2002; Terness et al., 2002; Wang et al., 2014; Holmgaard et al., 2015). Despite pre-clinical evidence (Spranger et al., 2014; Triplett et al., 2018) and promising results in several Phase I/II clinical trials (Gibney et al., 2015; Gangadhar et al., 2017; Hamid et al., 2017; Daud et al., 2018; Mitchell et al., 2018; Zakharia et al., 2018), an IDO-1 inhibitor failed to show clinical benefit in a Phase III clinical trial (Long et al., 2019). A treatment can be quite effective in a specific subset without being effective in the whole cancer type, e.g., Herceptin is very effective in HER2+ breast cancer but not effective in breast cancer overall (Hudziak et al., 1989), so it is important to identify the subsets of cancers that may benefit from IDO-1 inhibitors. Since it is unknown whether high *IDO-1* expression is a sufficient predictor of response to IDO-1 inhibitors or whether high expression of other genes in the IDO pathway (*IDO-2* and *TDO-2*) is also necessary for response to IDO-1 inhibitors, in this study, we looked for the genomic and immunologic correlates of IDO pathway expression in cancer using the Cancer Genome Atlas (TCGA) dataset.

## Materials and Methods

The mRNA expression data of tumors from TCGA was downloaded from the Broad Genome Data Analysis Center^1^ and the TCGA Data Portal.^2^ The data was median adjusted and log_2_ transformed as previously described (Panda et al., 2018b). The breast cancer samples were classified into clinical subtypes (ER+/HER2−, ER−/HER2−, and HER2+) using the focal copy number data of *ERBB2* and the mRNA expression data of *ESR1* obtained from the Broad Genome Data Analysis Center, and the three clinical subtypes were analyzed separately. Hyper-mutation status (Panda et al., 2017), Epstein-Barr virus (EBV) status (Cancer Genome Atlas Research Network, 2014), Hepatitis B and C virus (HBV, HCV) status (Cancer Genome Atlas Research Network, 2017), and *ERV3-2* expression levels (Rooney et al., 2015) were obtained from recently published studies. Human papilloma virus (HPV) status was compiled from the auxiliary clinical files available in the TCGA Data Portal. Wilcoxon rank-sum test was used for all pairwise comparisons and Spearman Rho was used in all correlation analysis. All *p*-values are from two-sided tests, multiple hypothesis testing corrections were performed using the Benjamini-Hochberg method, and false discovery rate (FDR) < 0.05 was used as the threshold for statistical significance.

## Results

### Correlation Between the IDO Pathway Expression and *CD8A* Expression

**Figure 1:**
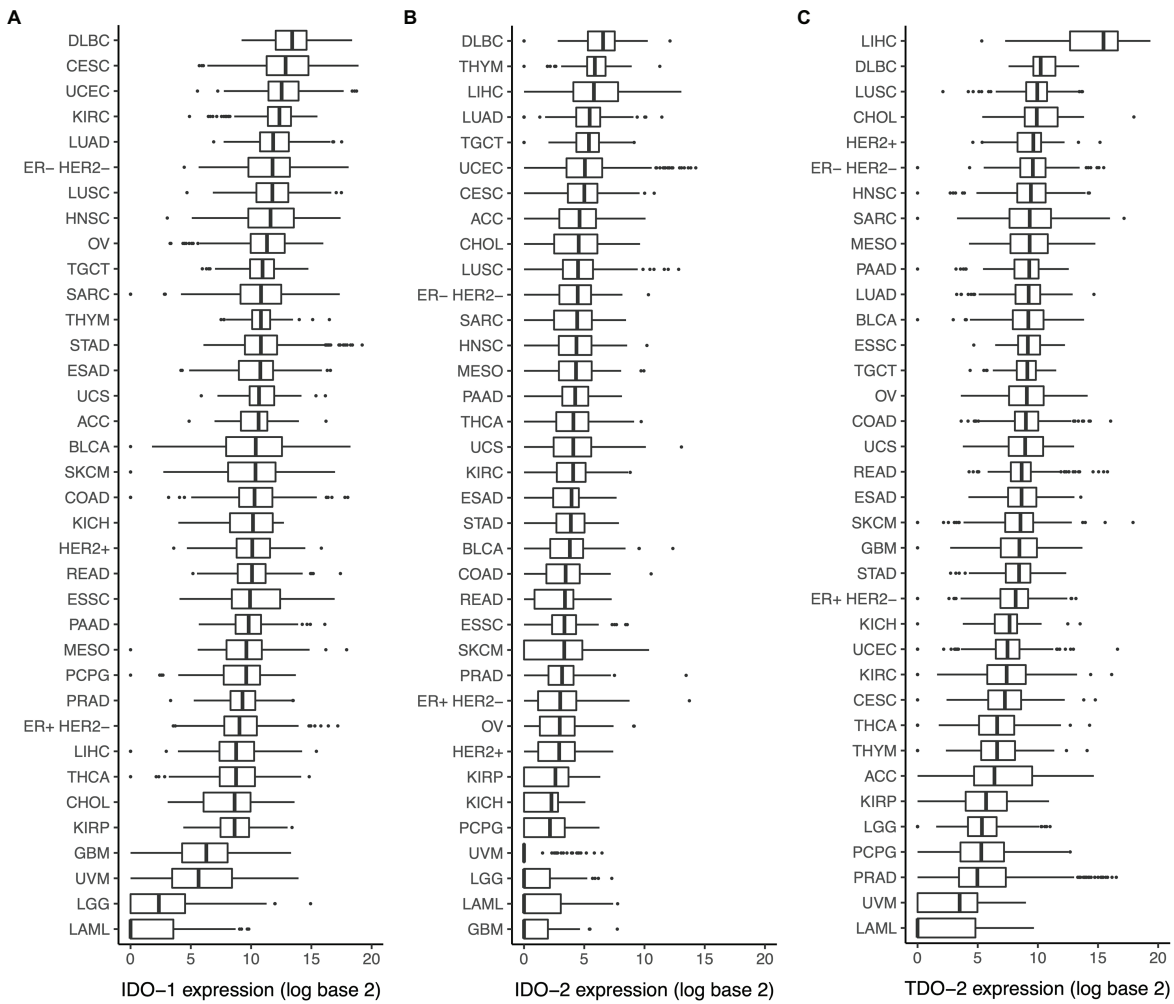
Expression of indoleamine 2,3-dioxygenase (IDO) pathway genes in various cancer types. Expression of **(A)** *IDO-1*, **(B)** *IDO-2*, and **(C)** *TDO-2* in various cancer types. Cancer type acronyms are standard the Cancer Genome Atlas (TCGA) abbreviations (https://gdc.cancer.gov/resources-tcga-users/tcga-code-tables/tcga-study-abbreviations). ESAD, esophageal adenocarcinoma; ESSC, esophageal squamous cell carcinoma.

Analysis of the TCGA dataset showed a wide range of *IDO-1* expression among different cancer types, with the highest median expression in diffuse large B-cell lymphoma and the lowest median expression in acute myeloid leukemia (Figure 1A). *IDO-2* (Figure 1B) and *TDO-2* (Figure 1C) expression showed a similar pattern, with high expression in diffuse large B-cell lymphoma and low expression in acute myeloid leukemia.

**Figure 2:**
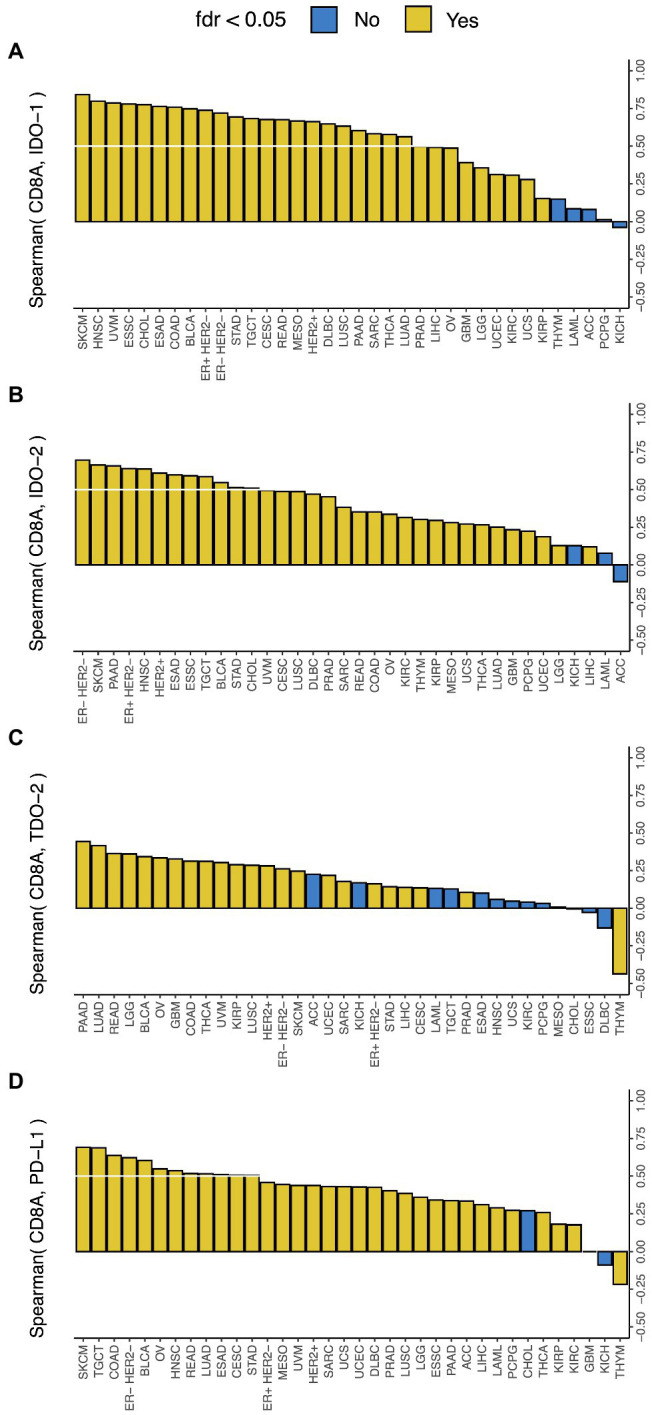
Correlation between IDO pathway expression and *CD8A* expression. Correlation between expressions of *CD8A* and **(A)** *IDO-1*, **(B)** *IDO-2*, **(C)** *TDO-2*, and **(D)** *PD-L1* (the white lines mark Spearman Rho = 0.5). Cancer type acronyms are standard TCGA abbreviations (https://gdc.cancer.gov/resources-tcga-users/tcga-code-tables/tcga-study-abbreviations). FDR, false discovery rate. ESAD, esophageal adenocarcinoma; ESSC, esophageal squamous cell carcinoma.

CD8^+^ T-cell infiltration in tumor is associated with local immune activation and response to immune checkpoint therapy (Tumeh et al., 2014), and PD-L1 expression is a marker of response to PD-1 blockade (Khunger et al., 2017). In this study, we found that *CD8A* expression, a known marker of CD8^+^ T-cell infiltration, had a much stronger correlation with *IDO-1* expression than *PD-L1* expression, a comparable correlation with *IDO-2* expression and *PD-L1* expression, and a much weaker correlation with *TDO-2* expression than *PD-L1* expression (Figure 2). This suggests that the presence of CD8^+^ T-cell infiltration in tumor is very strongly correlated with *IDO-1* expression, strongly correlated with *IDO-2* expression, but not so strongly correlated with *TDO-2* expression. *CD8A* expression was correlated with all three genes in 22 cancer types, and with *IDO-1* and *IDO-2* (but not *TDO-2*) in nine more cancer types, so CD8^+^ T-cell infiltration is likely a widespread predictor of IDO pathway over-expression. These results are consistent with and substantially extend the finding of a recent study (Feng et al., 2020) that observed a strong correlation between *IDO-1* expression and T-cell infiltration in breast and gynecologic cancers.

### Association Between the IDO Pathway Expression and Tumor Mutational Burden

We recently showed that in eight solid cancer types, the tumors above a non-synonymous mutation burden threshold (iCAM+) show RNA-seq based evidence of immune activation and immune checkpoint pathway upregulation in the TCGA dataset (Panda et al., 2017, 2020), and are more sensitive to immune checkpoint blockade in publicly available datasets of multiple cancer types (Panda et al., 2017) compared with the tumors below this threshold (iCAM−).

**Figure 3:**
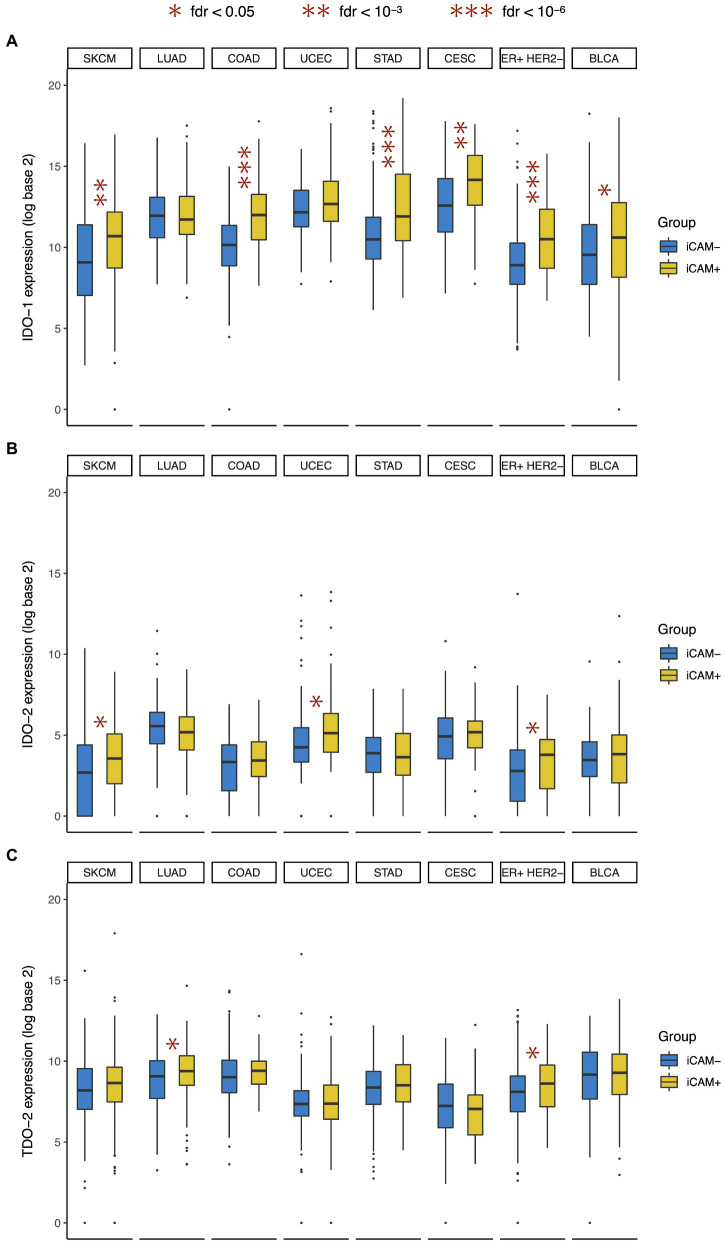
Association between IDO pathway expression and tumor mutational burden. Expression of **(A)** *IDO-1*, **(B)** *IDO-2*, and **(C)** *TDO-2* in high mutation burden (iCAM+) and low mutation burden (iCAM−) tumors. Cancer type acronyms are standard TCGA abbreviations (https://gdc.cancer.gov/resources-tcga-users/tcga-code-tables/tcga-study-abbreviations). FDR, false discovery rate. ^*^fdr < 0.05, ^**^fdr < 10^-3^, and ^***^fdr < 10^-6^.

In this study, we found that compared to the tumors with low mutation burden (iCAM−), the tumors with high mutation burden (iCAM+) had significantly higher *IDO-1* expression in most (6/8) cancer types (Figure 3A), but significantly higher *IDO-2* expression in only a minority (3/8) of cancer types (Figure 3B), and significantly higher *TDO-2* expression in very few (2/8) cancer types (Figure 3C). This suggests that high mutation burden is often associated with *IDO-1* over-expression, but only rarely associated with *IDO-2* over-expression, and very rarely associated with *TDO-2* over-expression. High mutation burden was associated with over-expression of all three genes in ER+ HER2− breast cancer, and over-expression of *IDO-1* and *IDO-2* (but not *TDO-2*) in skin melanoma. These results are consistent with and substantially extend the finding of a recent study (Feng et al., 2020) that observed a significant correlation between tumor mutational burden and *IDO-1* expression in breast and cervical cancer.

### IDO Pathway Expression in Virally Mediated Tumors

Virally mediated tumors, like EBV+ gastric cancer (Grogg et al., 2003; Chiaravalli et al., 2006; Panda et al., 2018b) and HPV+ cervical cancer (Varn et al., 2018) and head and neck squamous cell cancer (Varn et al., 2018), are associated with immune activation and immune checkpoint pathway upregulation (Panda et al., 2020). EBV+ tumors also respond to PD-1 blockade in gastric cancer (Panda et al., 2018b), NK/T-cell lymphoma (Kwong et al., 2017), and Hodgkin’s lymphoma (Carbone et al., 2018).

**Figure 4:**
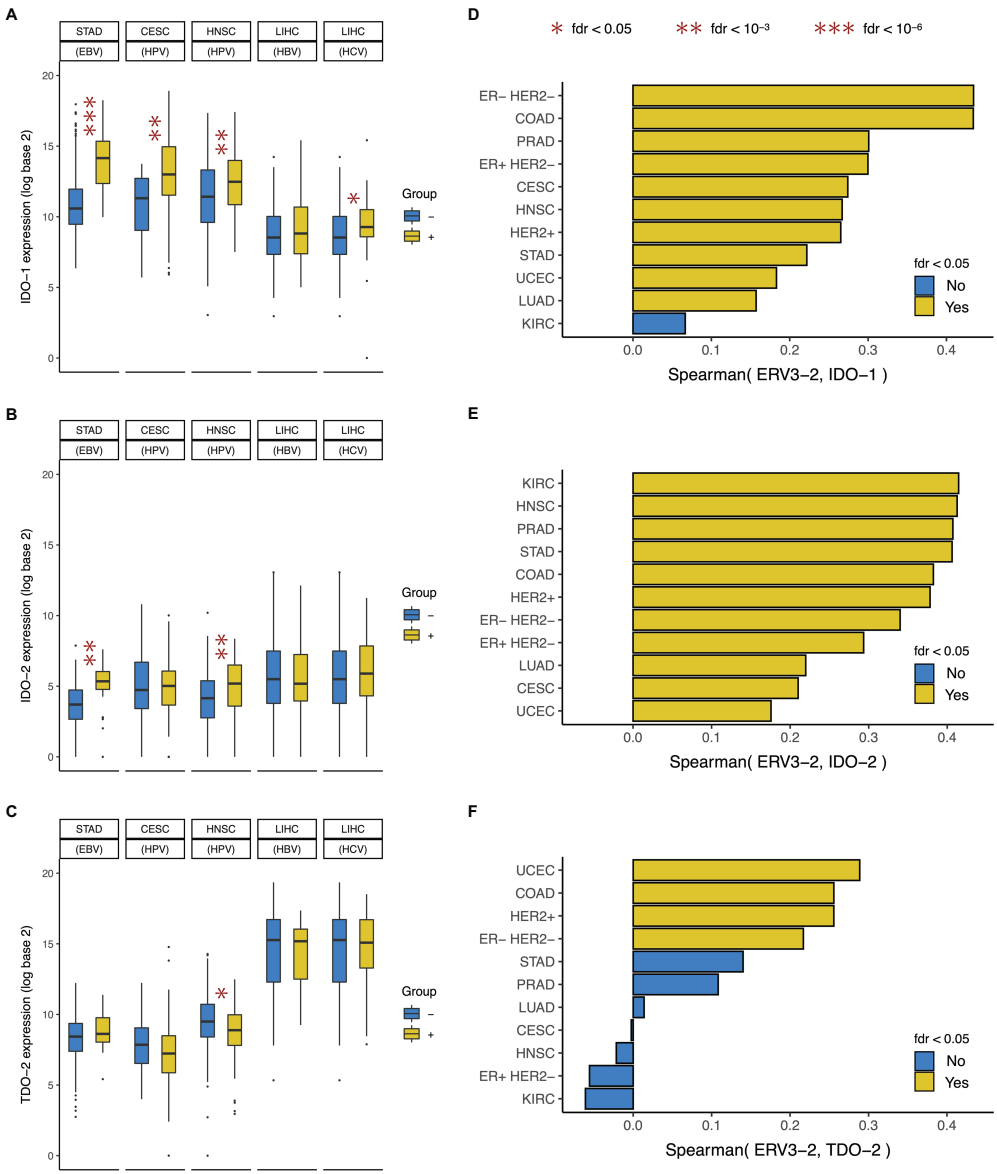
Association between IDO pathway expression and viral expression in tumor. Expression of **(A)** *IDO-1*, **(B)** *IDO-2*, and **(C)** *TDO-2* in tumors positive and negative for Epstein-Barr virus (EBV), Human papilloma virus (HPV), Hepatitis B virus (HBV), and Hepatitis C virus (HCV). Correlation between expressions of *ERV3-2* and **(D)** *IDO-1*, **(E)** *IDO-2*, and **(F)** *TDO-2*. Cancer type acronyms are standard TCGA abbreviations (https://gdc.cancer.gov/resources-tcga-users/tcga-code-tables/tcga-study-abbreviations). In liver cancer, the negative group only includes tumors negative for both HBV and HCV (i.e., HBV− HCV−) and does not include tumors that are HBV+ HCV− or HBV− HCV+. FDR, false discovery rate. ^*^fdr < 0.05, ^**^fdr < 10^-3^, and ^***^fdr < 10^-6^.

In this study, we found that EBV+ gastric cancer, HPV+ cervical and head-neck squamous cell cancer, and HCV+ liver cancer (but not HBV+ liver cancer) showed over-expression of *IDO-1* (Figure 4A), but only EBV+ gastric cancer and HPV+ head and neck squamous cell cancer showed over-expression of *IDO-2* (Figure 4B). Virally mediated tumors did not show over-expression of *TDO-2*, in fact HPV+ head and neck squamous cell cancer showed under-expression of *TDO-2* (Figure 4C). This suggests that exogenous viral infection in tumor (e.g., EBV, HPV, and HCV) is associated with the over-expression of *IDO-1* and sometimes *IDO-2* but not *TDO-2*. These results are consistent with and extend the findings of previous studies that reported *IDO-1* over-expression in EBV+ nasopharyngeal cancer (Liu et al., 2014), HPV+ cervical (Kobayashi et al., 2008) and head-neck squamous cell cancer (Sailer et al., 2019), and chronic HCV infected liver (Larrea et al., 2007).

### Correlation Between the IDO Pathway Expression and Expression of Potentially Immunogenic ERV

Expression of endogenous retrovirus (ERV) is a potential mechanism of immune activation (Hurst and Magiorkinis, 2015), and we recently showed that the expression of *ERV3-2* in tumor is correlated with immune activation and immune checkpoint pathway upregulation in 11 solid cancer types (Panda et al., 2018a) and response to PD-1 blockade in clear-cell renal cancer (Panda et al., 2018a).

In this study, we found that *ERV3-2* expression was correlated with *IDO-1* expression in most (10/11) of these cancer types (Figure 4D), *IDO-2* expression in all (11/11) of these cancer types (Figure 4E), and *TDO-2* expression in only a few (4/11) of these cancer types (Figure 4F). Interestingly, *ERV3-2* expression was often more strongly correlated with *IDO-2* expression than *IDO-1* expression. These results suggest that the expression of potentially immunogenic ERV is associated with the over-expression of both *IDO-1* and *IDO-2*, but rarely *TDO-2*. *ERV3-2* expression was correlated with all three genes in ER− HER2− and HER2+ breast, colon, and endometrial cancers; and *IDO-1* and *IDO-2* (but not *TDO-2*) in ER+ HER2− breast, gastric, lung, prostate, cervical, and head-neck squamous cell cancers.

### Association Between the IDO Pathway Expression and Patient Survival

Survival analysis using UALCAN (Chandrashekar et al., 2017) shows that high expression of *IDO-1* was often associated with worse prognosis (Supplementary Figure 1), such as in low-grade glioma, papillary renal cancer, pancreatic cancer, endometrial cancer, and uveal melanoma. However, skin melanoma was an exception to this, where high expression of *IDO-1* was associated with better prognosis (Supplementary Figure 1). Like *IDO-1*, high expression of *TDO-2* was also associated with worse prognosis (Supplementary Figure 2), such as in low-grade glioma, clear cell renal cancer, papillary renal cancer, acute myeloid leukemia, mesothelioma, and testicular germ cell tumor. Unlike *IDO-1* and *TDO-2*, high expression of *IDO-2* was often associated with better prognosis (Supplementary Figure 3). While high expression of *IDO-2* was associated with worse prognosis in three cancer types, namely clear cell renal cancer, papillary renal cancer, and testicular germ cell tumor, it was associated with better prognosis in six cancer types, namely cervical cancer, head-neck squamous cell cancer, mesothelioma, pancreatic cancer, sarcoma, and skin melanoma (Supplementary Figure 3). These results suggest that high expression of *IDO-1* (except in skin melanoma) and *TDO-2* werse often associated with worse prognosis, while high expression of *IDO-2* was often associated with better prognosis (with a few exceptions).

## Discussion

CD8^+^ T-cell infiltration in tumor (Tumeh et al., 2014), high tumor mutational burden (Mehnert et al., 2016; Panda et al., 2017), exogenous viral infection in tumor (Kwong et al., 2017; Carbone et al., 2018; Panda et al., 2018b), and expression of endogenous retrovirus in tumor (Panda et al., 2018a) are associated with immune activation and checkpoint pathway upregulation in many cancer types, and response to immune checkpoint blockade in several cancer types.

In this study, we found that *CD8A* expression, a marker of CD8^+^ T-cell infiltration in tumor, was much more strongly correlated with *IDO-1* expression than *PD-L1* expression, a marker of response to PD-1 blockade (Khunger et al., 2017); and high tumor mutational burden, exogenous viral infection in tumor, and expression of endogenous retrovirus in tumor were associated with *IDO-1* over-expression in most cancer types. These results are consistent with and substantially extend the finding of a recent study (Feng et al., 2020) that found *IDO-1* expression to be correlated with tumor mutational burden in breast and cervical cancer, and T-cell infiltration in breast and gynecologic cancers; and previous studies that reported *IDO-1* over-expression due to exogenous viral infection in EBV+ nasopharyngeal cancer (Liu et al., 2014), HPV+ cervical (Kobayashi et al., 2008) and head-neck squamous cell cancer (Sailer et al., 2019), and chronic HCV infected liver (Larrea et al., 2007).

*CD8A* expression showed a comparable level of correlation with *IDO-2* expression and *PD-L1* expression, and while high tumor mutational burden and exogenous viral infection in tumor were associated with *IDO-2* over-expression in only a minority of cancer types, expression of endogenous retrovirus in tumor was correlated with *IDO-2* expression even more strongly than *IDO-1* expression.

*CD8A* expression showed much weaker correlation with *TDO-2* expression than *PD-L1* expression, and high tumor mutational burden and endogenous retroviral expression in tumor were rarely associated with *TDO-2* expression, while exogenous viral infection in tumor was not at all associated with *TDO-2* expression. In fact, HPV+ head and neck squamous cell cancer showed under-expression of *TDO-2*.

Overall, our results show that tumors with strong CD8+ T-cell infiltration, or high mutation burden, or exogenous viral infection, or endogenous retroviral expression have high *IDO-1* expression in most cancer types, high *IDO-2* expression in many cancer types, but high *TDO-2* expression in only a few cancer types. These results may have important implications for guiding development clinical trials of IDO-1 inhibitors.

It is unknown whether high *IDO-1* expression is a sufficient predictor of response to IDO-1 inhibitors or whether high expression of other genes in the IDO pathway (*IDO-2* and *TDO-2*) is also necessary for response to IDO-1 inhibitors. *CD8A* expression is correlated with all three genes in 22 cancer types, and with *IDO-1* and *IDO-2* (but not *TDO-2*) in nine more cancer types, so CD8^+^ T-cell infiltration is likely a widespread predictor of IDO pathway over-expression. High mutational burden in ER+ HER2− breast cancer, and *ERV3-2* expression in ER− HER2− and HER2+ breast, colon, and endometrial cancers are also associated with over-expression of all three IDO pathway genes, so these features may predict benefit from IDO-1 inhibitors. High mutational burden in skin melanoma, exogenous viral infection in gastric (EBV) and head-neck squamous cell (HPV) cancers, and *ERV3-2* expression in ER+ HER2− breast, gastric, lung, prostate, cervical, and head-neck squamous cell cancers are associated with over-expression of *IDO-1* and *IDO-2* (but not *TDO-2*), but it is unclear whether that is sufficient for response to IDO-1 inhibitors.

Despite pre-clinical evidence (Spranger et al., 2014; Triplett et al., 2018) and promising results in several Phase I/II clinical trials (Gibney et al., 2015; Gangadhar et al., 2017; Hamid et al., 2017; Daud et al., 2018; Mitchell et al., 2018; Zakharia et al., 2018), an IDO-1 inhibitor failed to show clinical benefit in a Phase III clinical trial in melanoma (Long et al., 2019). While this Phase III clinical trial in melanoma was not restricted to tumors with high mutation burden and included tumors with low mutation burden, majority of melanoma cases tend to have high mutation burden (Panda et al., 2017). This might suggest that even the high mutation burden subset of melanoma, which shows over-expression of both *IDO-1* and *IDO-2* but not *TDO-2*, does not respond to IDO-1 inhibitors and over-expression of all three genes are necessary for response to IDO-1 inhibitors. Further research is necessary to test whether this is indeed the case.

## Data Availability Statement

The original contributions presented in the study are included in the article/Supplementary Material; further inquiries can be directed to the corresponding author.

## Author Contributions

AP: idea development, all analysis, all figures, manuscript writing, editing, and formatting. SG: idea development, interpretation of results, and editing. All authors contributed to the article and approved the submitted version.

### Conflict of Interest

SG consulted for Novartis, Roche, Foundation Medicine, Foghorn Therapeutics, and Inspirata; Owns equity in and has licensed patents to Inspirata; Spouse is an employee of Merck and has equity in Merck.

The remaining author declares that the research was conducted in the absence of any commercial or financial relationships that could be construed as a potential conflict of interest.

The handling editor declared a past co-authorship with the authors AP and SG.

## Footnotes

^1^http://gdac.broadinstitute.org

^2^https://tcga-data.nci.nih.gov/docs/publications/tcga/

## Supplementary Material

Supplementary figure 1 Association between *IDO-1* expression and patient survival.

Supplementary figure 2 Association between *TDO-2* expression and patient survival.

Supplementary figure 3 Association between *IDO-2* expression and patient survival.
