# Supplementary figures and images for: Genomic and Immunologic Correlates of Indoleamine 2,3-Dioxygenase Pathway Expression in Cancer
Source: Front Genet. 2021 Jul 22;12:706435. doi: 10.3389/fgene.2021.706435 (PMC8340027; doi:10.3389/fgene.2021.706435)

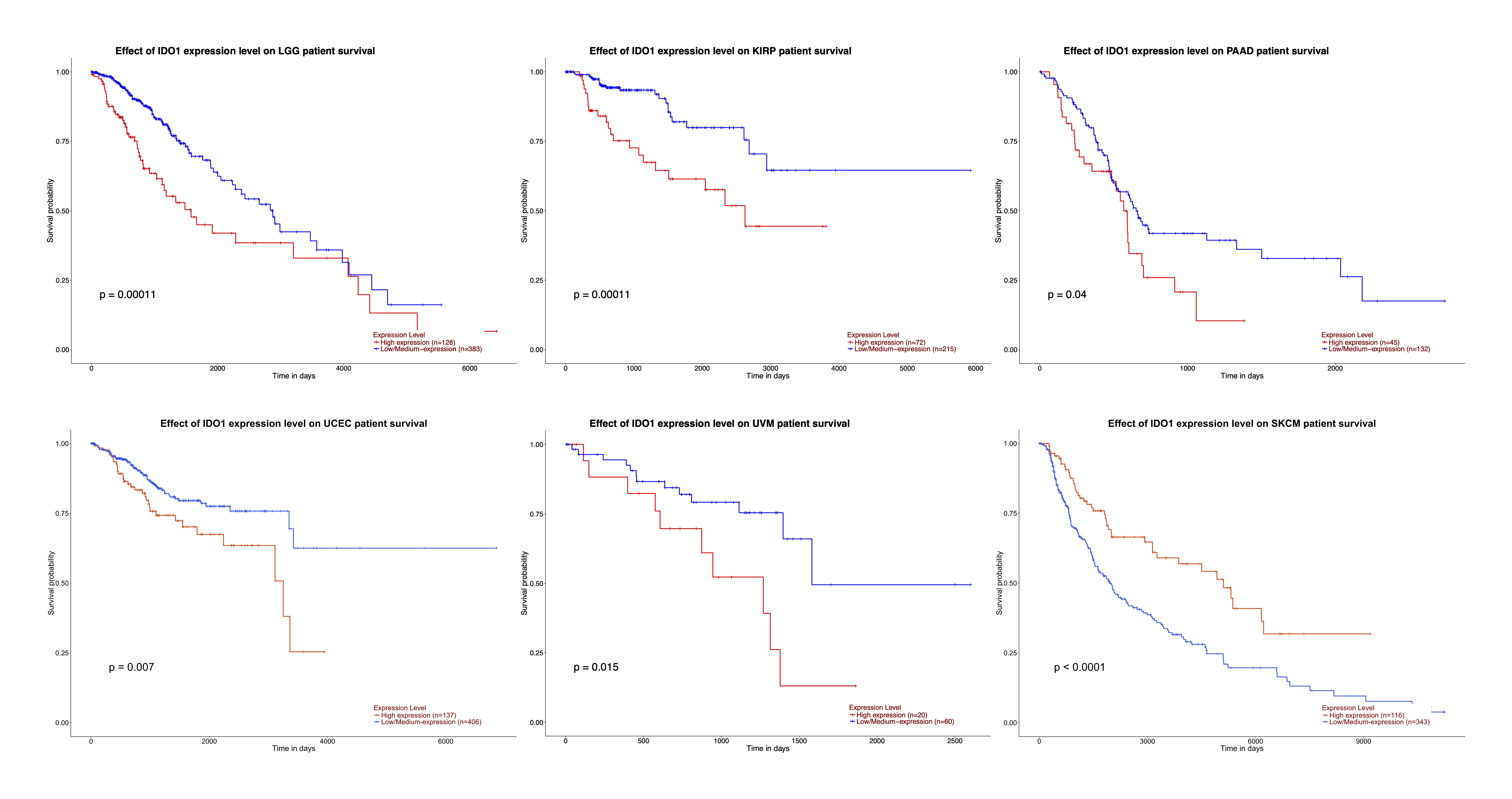

Supplement: Supplementary figure 1 — Association between IDO-1 expression and patient survival. [file Image_1.JPEG]

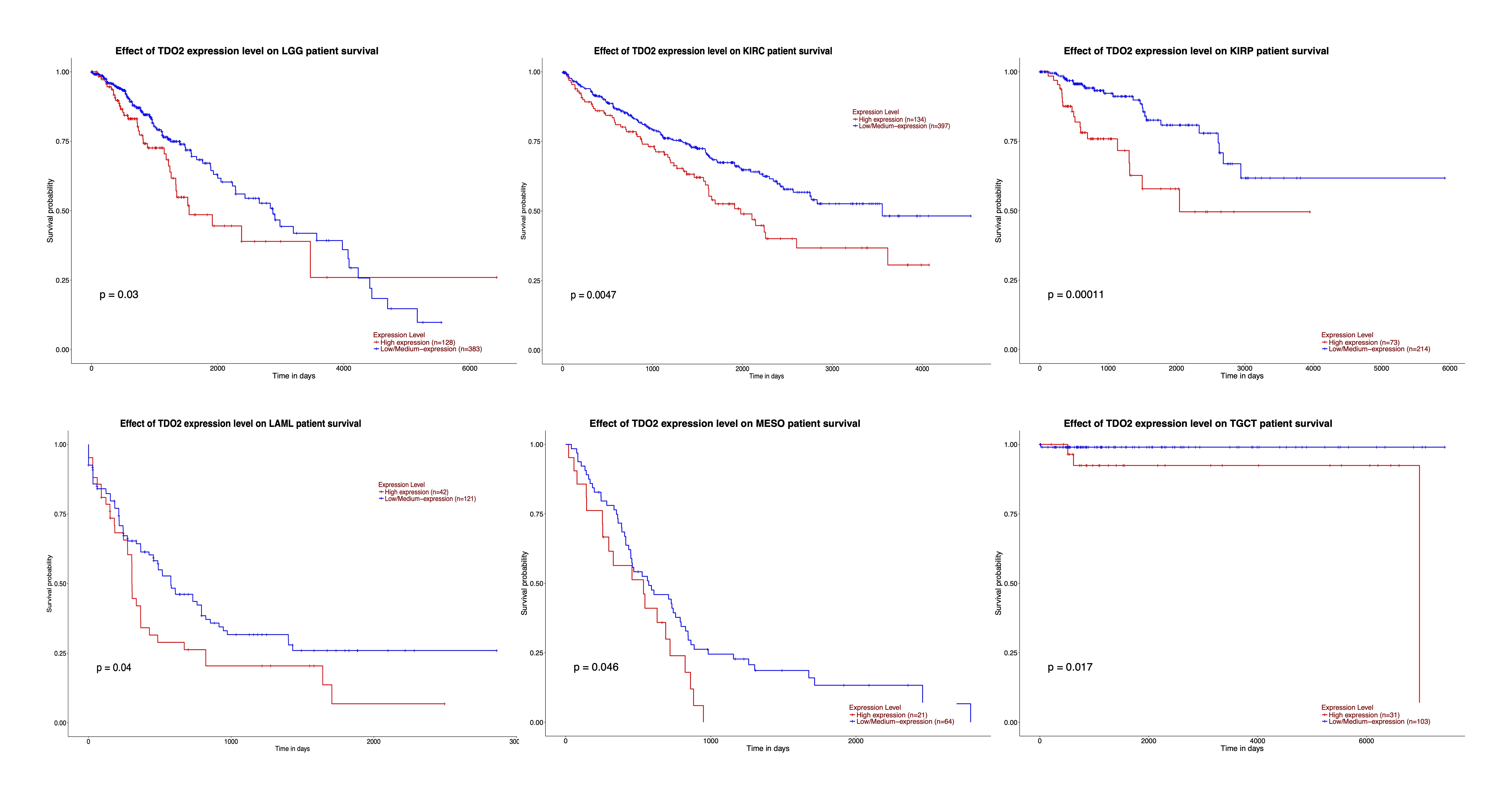

Supplement: Supplementary figure 2 — Association between TDO-2 expression and patient survival. [file Image_2.JPEG]

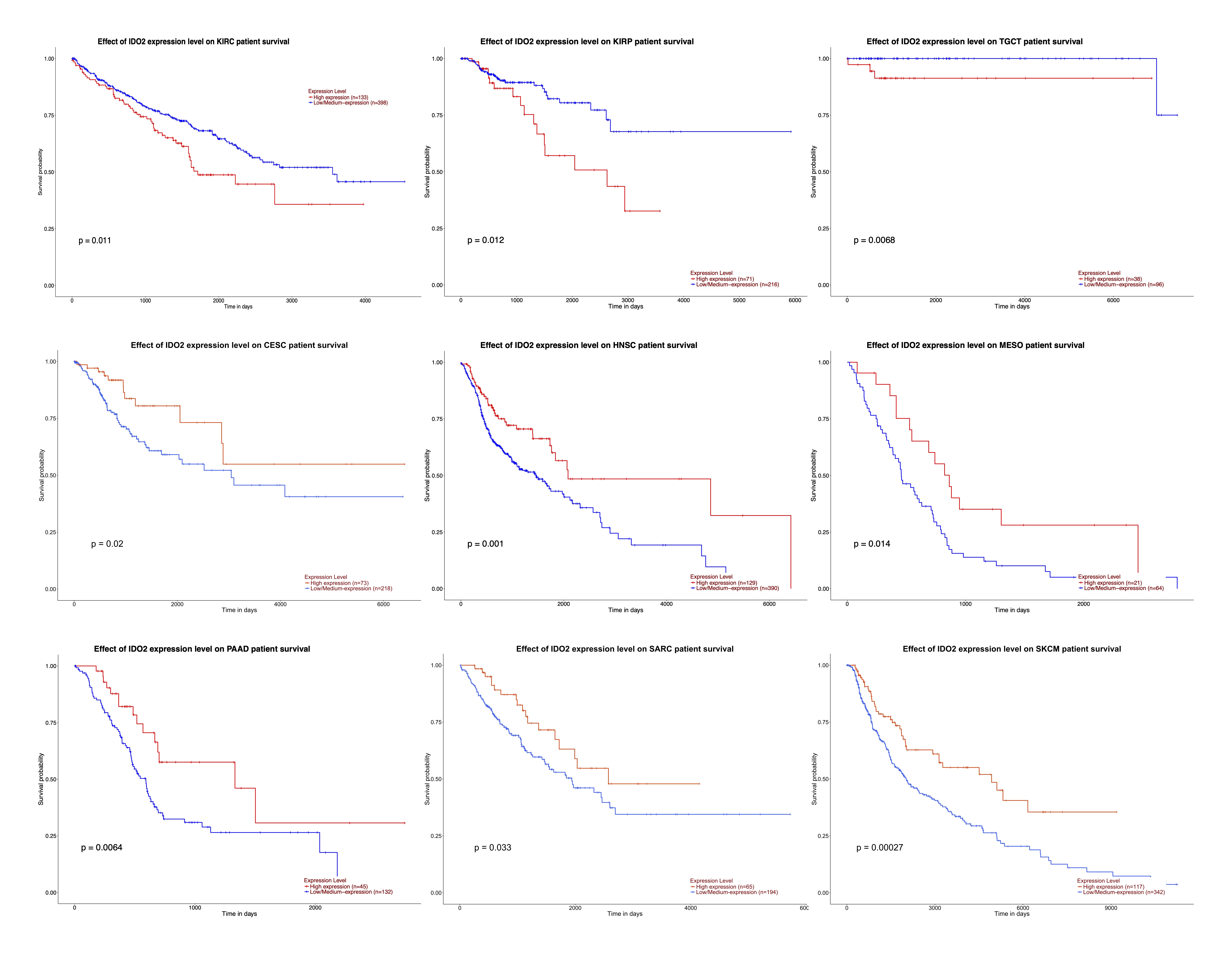

Supplement: Supplementary figure 3 — Association between IDO-2 expression and patient survival. [file Image_3.JPEG]
